# Perspectives on sustainability among surgeons: findings from the SAGES-EAES sustainability in surgical practice task force survey

**DOI:** 10.1007/s00464-024-11137-7

**Published:** 2024-08-19

**Authors:** Tejas S. Sathe, Adnan Alseidi, Vittoria Bellato, Amir Ashraf Ganjouei, Laleh Foroutani, Ryan P. Hall, Oleksii Potapov, Ricardo J. Bello, Shaneeta M. Johnson, Stefania Marconi, Nader Francis, Paul Barach, Manuel Sanchez-Casalongue, Sheetal Nijhawan, Wendelyn M. Oslock, Benjamin Miller, Sarah Samreen, Jimmy Chung, Nana Marfo, Bright Huo, Robert B. Lim, Jonathan Vandeberg, Yewande R. Alimi, Andrea Pietrabissa, Alberto Arezzo, Maximos Frountzas, Miran Rems, M. M. M. Eussen, N. D. Bouvy, Patricia Sylla

**Affiliations:** 1 Department of Surgery, University of California San Francisco, San Francisco, USA; 2 Department of Minimally Invasive Surgery, University Hospital of Rome Tor Vergata, Rome, Italy; 3 Department of Surgery, Tufts Medical Center, Boston, USA; 4 Department of General Surgery, College of Medicine, University of Rzeszow, Rzeszow, Poland; 5 Department of Surgery, Medical College of Wisconsin, Milwaukee, USA; 6 Department of Surgery, Morehouse School of Medicine, 720 Westview Drive, Atlanta, GA 30310 USA; 7 Department of Civil Engineering and Architecture, University of Pavia, Pavia, Italy; 8 IRCCS Policlinico San Matteo Foundation, Pavia, Italy; 9 Griffin Institute, London, UK; 10 Thomas Jefferson University School of Medicine, Philadelphia, USA; 11 Department of General Surgery, Imperial College London, London, UK; 12 Department of Surgery, Clinica San Camilo, Buenos Aires, Argentina; 13 Department of Surgery, Sharon Regional Medical Center, Sharon, PA USA; 14 Department of Surgery, University of Alabama Birmingham, Birmingham, AL USA; 15 Department of Quality, Birmingham Veterans Affairs Medical Center, Birmingham, AL USA; 16 Cleveland Clinic Foundation, Cleveland, OH USA; 17 Division of Minimally Invasive Surgery, University of Texas Medical Branch, Galveston, TX USA; 18 Adventus Health Partners, Cincinnati, OH USA; 19 Ross University School of Medicine, Miramar, FL USA; 20 Department of General Surgery, McMaster University, Ontario, CA USA; 21 Department of Surgery, Atrium Carolinas Medical Center, Wake Forest University, Charlotte, NC USA; 22 Department of Surgery, Mayo Clinic Florida, Jacksonville, FL USA; 23 Department of Surgery, Medstar Georgetown University Hospital, Washington, DC USA; 24 Department of General Surgery, University of Pavia, Pavia, Italy; 25 Department of Surgical Sciences, University of Turin, Turin, Italy; 26 First Propaedeutic Department of Surgery, Hippocration General Hospital, National and Kapodistrian University of Athens, Athens, Greece; 27 Department of General and Abdominal Surgery, General Hospital Jesenice, Jesenice, Slovenia; 28 Department of Surgery, Maastricht University Medical Center, Maastricht, The Netherlands; 29 NUTRIM School of Nutrition and Translational Research in Metabolism, Maastricht University, Maastricht, The Netherlands; 30 Division of Colon and Rectal Surgery, Mount Sinai Health System, New York, NY USA

**Keywords:** Sustainability, Operating room waste, Decarbonization, Climate change, Carbon footprint, Minimally invasive surgery

## Abstract

**Background:**

Surgical care significantly contributes to healthcare-associated greenhouse gas emissions (GHG). Surgeon attitudes about mitigation of the impact of surgical practice on environmental sustainability remains poorly understood. To better understand surgeon perspectives globally, the Society of American Gastrointestinal and Endoscopic Surgeons and the European Association for Endoscopic Surgery established a joint Sustainability in Surgical Practice (SSP) Task Force and distributed a survey on sustainability.

**Methods:**

Our survey asked about (1) surgeon attitudes toward sustainability, (2) ability to estimate the carbon footprint of surgical procedures and supplies, (3) concerns about the negative impacts of sustainable interventions, (4) willingness to change specific practices, and (5) preferred educational topics and modalities. Questions were primarily written in Likert-scale format. A clustering analysis was performed to determine whether survey respondents could be grouped into distinct subsets to inform future outreach and education efforts.

**Results:**

We received 1024 responses, predominantly from North America and Europe. The study revealed that while 63% of respondents were motivated to enhance the sustainability of their practice, less than 10% could accurately estimate the carbon footprint of surgical activities. Most were not concerned that sustainability efforts would negatively impact their practice and showed readiness to adopt proposed sustainable practices. Online webinars and modules were the preferred educational methods. A clustering analysis identified a group particularly concerned yet willing to adopt sustainable changes.

**Conclusion:**

Surgeons believe that operating room waste is a critical issue and are willing to change practice to improve it. However, there exists a gap in understanding the environmental impact of surgical procedures and supplies, and a sizable minority have some degree of concern about potential adverse consequences of implementing sustainable policies. This study uniquely provides an international, multidisciplinary snapshot of surgeons’ attitudes, knowledge, concerns, willingness, and preferred educational modalities related to mitigating the environmental impact of surgical practice.

**Supplementary Information:**

The online version contains supplementary material available at 10.1007/s00464-024-11137-7.

The harmful effects of climate change are increasingly evident. Issues such as poor air quality, reduced access to clean drinking water, increasing rates of heat-related mortality, disruption in supply chains, exacerbation of chronic health conditions, and mass migration are all exacerbated by an increasingly warming planet [1, 2]. Moreover, it is now well accepted that human-generated carbon emissions are directly responsible for this warming. As signatories of the Paris Climate Agreement, the United States and European countries, among 195 others, recognize the need to limit the rise in global temperatures to no more than two degrees Celsius above pre-industrial levels [3]. Through recent legislation, the United States and the European Union have committed to operationalize the Paris Agreement’s targets by halving net carbon emissions by 2030 and achieving net zero emissions by 2050 [4, 5]. Such ambitious decarbonization will require the cooperation of all energy-intensive sectors of the economy.

There is a growing acknowledgment of the role that the health sector plays in generating emissions and contributing to climate change. The health sector accounts for about 4% of global GHG emissions, and over 8% of U.S. GHG emissions [6]. To address this, governments and institutions are establishing health sector specific emissions goals that are aligned with national and international efforts. For example, the United States Department of Health and Human Services released a voluntary climate pledge that asks hospital systems to halve emissions by 2030 and achieve net zero emissions by 2050, in line with the Paris Climate Agreement [7]. Over 100 hospitals have signed thus far. In 2023, the Joint Commission, which oversees hospital accreditation, released guidelines for hospitals to achieve sustainable healthcare certification, at present a voluntary designation [8]. Among health-associated emissions, surgery is one of the largest individual contributors. Surgical care, in particular, is responsible for up to 30% of hospital waste production [9–11]. As such, there is a need for surgeons to contribute to the improvement of healthcare sustainability.

The fact that surgery, and minimally invasive surgery in particular, is a resource intensive and sometimes wasteful discipline may not be a surprise to any of its practitioners. However, surgeons are only beginning to understand how such waste contributes to increased emissions. Many institutions, organizations, and societies have attempted to educate surgeons on this issue and provide resources to improve sustainability [12, 13]. Over the past decade, surgeons across many disciplines have quantified emissions from several aspects of surgical care and posited how to reduce them [14, 15]. Many have even implemented interventions within their departments and institutions [16]. Unfortunately, these efforts have not yet been coordinated to achieve a tangible reduction in health-associated carbon emissions. To the contrary, the contribution of the health sector to U.S. GHG emissions has increased over the past decade [10].

A growing body of literature aims to understand how surgeons feel about improving the sustainability of their practice. Results of these studies tend to show that surgeons recognize the reality and consequences of climate change and are motivated to implement solutions, though support for specific remedies varies [17–24]. Furthermore, the majority of these studies are limited to one institution, subspecialty or country. What results is a fractured account of surgeons’ perspectives on sustainability and a lack of consensus on how to tangibly achieve it. In addition to better understanding surgeon attitudes across various geographies and practices, what is needed is a process to identify surgeons who are skeptical but persuadable about improving sustainability and designing educational efforts that are tailored to this subgroup’s constraints.

To address this, SAGES and EAES jointly established a SSP Task Force with three aims: (1) to understand the current state of surgeon knowledge and attitudes about climate change and sustainability, (2) to educate surgeons on the environmental impact of their practices, how to mitigate this impact, and why such efforts are worthwhile, and (3) to meaningfully reduce the carbon impact of surgical care, through recommendations, guidelines, and industry partnerships (Fig. 1) [25]. In this paper, we present the results of a sustainability survey designed to address the first aim. Specifically, we sought to measure surgeons’ general attitudes on sustainability, understanding of the environmental impact of surgery, concerns about implementing sustainable changes, willingness to implement specific changes, and preferences for educational modalities. Collectively, our results represent one of the first multidisciplinary and intercontinental assessments of surgeons’ overall perspectives on sustainability.

**Fig. 1:**
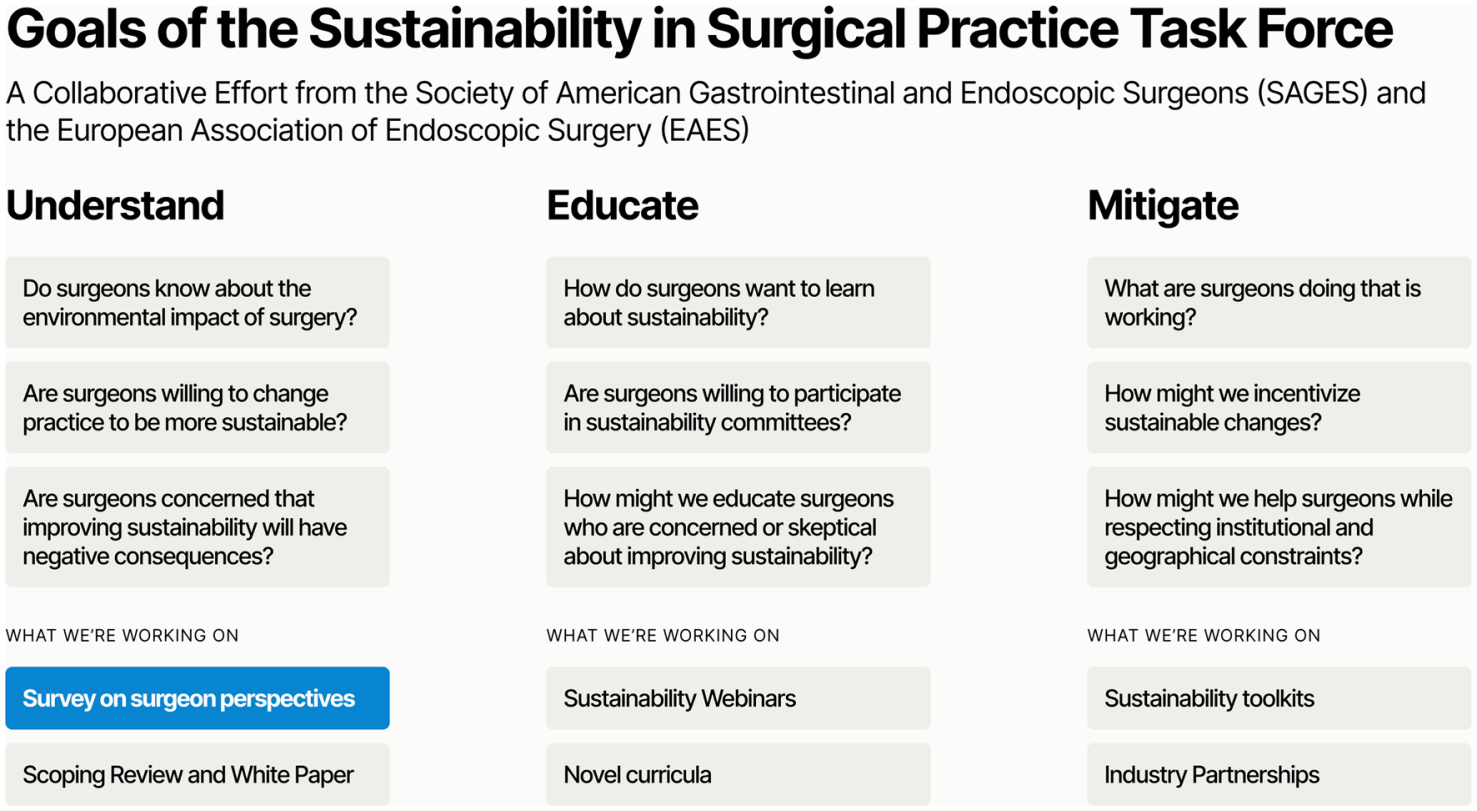
Schematic explaining the current survey study in the context of the broader aims of the SAGES-EAES Sustainability in Surgical Practice (SSP) task force

## Materials and methods

### Survey design

We performed a cross-sectional survey to understand surgeons’ perspectives on sustainability. Specifically, we wanted to answer the following five questions:

1. What are surgeons’ general attitudes toward the sustainability of surgical practice?
2. How well can surgeons estimate the carbon footprint of surgical procedures and supplies?
3. Are surgeons concerned that implementing sustainable changes will negatively impact their practice?
4. How willing are surgeons to make evidence-based sustainable changes?
5. What educational modalities and topics are most exciting to surgeons?

The survey was initially developed by members of the SAGES-EAES SSP Task Force using Qualtrics (Qualtrics International Inc, Seattle, WA). The survey was refined in an iterative process informed by two experienced survey methodologists. We subjected the survey to member checking and conducted cognitive interviews with Task Force members as well as outside content experts on sustainability. We then conducted additional beta testing with a broader convenience sample of graduate and medical students. The final version of the survey was approved by the SSP Task Force leadership as well as the boards of both surgical organizations. Our study protocol was approved by the University of California San Francisco Institutional Review Board (Protocol Number: 22-37453).

### Survey distribution

The survey was distributed to members of both SAGES and EAES via email distribution. Multiple email reminders were sent to members of both societies. In addition, the survey was distributed via social media (e.g., X, SAGES Facebook group) and the personal networks of member surgeons. We included surgeons, trainees, and medical students across the world and excluded incomplete responses. The survey was open from October 20, 2023 to January 1, 2024 (102 days).

### Analysis of categorical questions

Our questions related to general attitudes, current understanding, and concerns were based on a five-point Likert scale ranging from “not at all” to “extremely” with the following term based on the question (e.g., “not at all concerned” to “extremely concerned”). For questions related to surgeon willingness, we utilized a seven-point scale that included five points ranging from “not at all willing” to “extremely willing” as well as two additional options for “I am unable to do this” and “I am already doing this.”

For our analysis, we measured the proportion of respondents who chose the two or three most “pro-sustainability” responses as a measure of strong support for each item. Specifically, for questions related to general attitudes and education, we calculated the proportion of respondents who chose the two most positive responses (“quite” or “extremely”). For questions related to surgeon concerns, we calculated the proportion of respondents who were “not at all concerned” or “slightly concerned.” For questions related to surgeon willingness, we calculated the proportion of respondents who were “quite willing,” “extremely willing,” or “already doing” the proposed change.

For questions related to understanding preferred educational modalities, we used a ranked choice voting format where we allowed respondents to rank their preferred educational modalities and topics based on excitement. For these questions, we ranked responses using a Borda count. For example, if given five options, we gave a respondent five points for their first choice, four points for their second choice, three points for their third choice, two points for their fourth choice, and one point for their fifth choice and subsequently added point tallies for each option [26].

### Comparing results across demographic groups

We created three demographic comparison groups to see whether there were differences in responses between sub-populations. The first comparison group was: United States versus Europe versus Other Regions. The second comparison group was: trainees (including medical students, residents, and fellows) versus non-trainees. The third comparison group was: leadership (including chairs, vice chairs, division chiefs, program directors, associate program directors, and sustainability leads) versus respondents without specific leadership roles. For each comparison, we measured the proportion of respondents with a pro-sustainability position as per our previous analyses. We measured differences between groups using a chi-squared test.

### Cluster analysis

We hypothesized that our respondents may comprise several “phenotypes” with regards to sustainability. For example, is there a group of surgeons who are especially willing to make sustainable changes? Is there another group that has a high degree of concern that such changes will negatively impact their practice? To understand this, we performed a cluster analysis. We did not choose the number of clusters or what questions inform the clustering a priori. For example, we did not pre-determine that the clustering analysis should separate individuals based on their level of concern about implementing specific changes. Rather, we used established techniques such as the elbow method to determine the optimal number of clusters, and an unsupervised K-means approach to determine the clusters themselves.

We then sought to understand whether there were differences in how individuals in each cluster responded to the survey. To do this, we created integer mappings of Likert responses for attitudes, understanding, and concerns (e.g., “not at all concerned,” “slightly concerned” = 2, “moderately concerned” = 3, “quite concerned” = 4, and “extremely concerned” = 5). For questions about willingness to implement sustainable practices, we used additional mappings (e.g., “I am not able to do this” = 0 and “I am already doing this” = 6). We then calculated the mean response score among respondents in each cluster for each question.

### Data and code

All formal analyses were performed using Python 3.10 (Python Software Foundation, Wilmington, DE) running on Microsoft Visual Studio Code with GitHub Copilot (Microsoft Corporation, Redmond, WA). We used medians and interquartile ranges to summarize continuous variables and proportions to summarize the categorical variables (such as those from Likert scale questions). In all analyses, a *p*-value of less than 0.05 was considered statistically significant.

## Results

### Responses

Overall, 1024 provided complete responses to our survey. As we solicited responses through social media and other modalities without a clear denominator, we were unable to calculate a response rate.

### Demographics

**Fig. 2:**
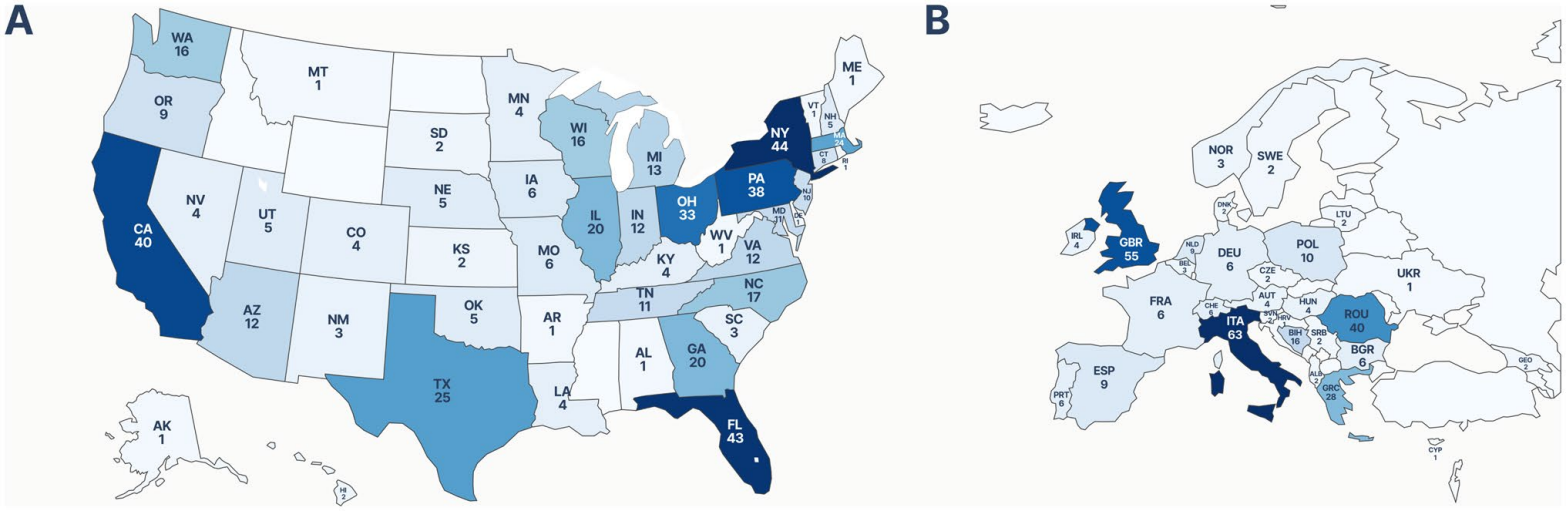
Number of respondents by **A** U.S. State and **B** European country. Darker shades indicate more respondents

Complete data on demographics is available in Table 1. Overall, respondents represented 80 countries. Notably, a slight majority of respondents (*n* = 564, 55%) were from North America, with 510 (50%) of all respondents from the United States (US), 36 (3.5%) from Canada, and 14 (1.4%) from Mexico. Meanwhile, 302 (29%) respondents were from Europe, with the greatest representation from Italy (*n* = 63, 6.1%), the United Kingdom (*n* = 55, 5.3%), and Romania (*n* = 40, 3.9%). Moreover, 116 (11%) of respondents were from Asia, with the greatest representation from India (*n* = 31, 3.0%), Japan (*n* = 19, 1.9%), and South Korea (*n* = 8, 0.8%). Finally, 21 (2.1%) participants were from South America, 16 (1.6%) were from Africa, and five (0.5%) were from Oceania. Though our respondents were predominantly from the US and Europe, we had strong representation across both geographies. Our respondents comprised 46 US states and 30 European countries (Fig. 2).

**Table 1:** Respondent demographics

Variable	Overall, *N* = 1024
Age, mean (SD)	44 (37, 54)
Years, mean (SD)	10 (3,20)
Practice setting size, *n* (%)	
Greater than 500 beds	407 (39.75%)
Between 100 and 500 beds	421 (41.11%)
Between 25 and 100 beds	151 (14.75%)
Fewer than 25 beds	45 (4.39%)
Hospital setting, *n* (%)	
Academic hospital	518 (50.59%)
Community or private teaching hospital	265 (25.88%)
Community or private non-teaching hospital	121 (11.82%)
Public access hospital	100 (9.77%)
Military and veterans hospital	20 (1.95%)
*Role, *n*	
Consultant/attending	830
Division chief	159
Program director/associate program director	149
Chair/vice-chair	142
Sustainability lead	16
Trainee	165
Resident/house staff	93
Fellow	68
Medical student	4
Other or blank	52
^a^Specialty, *n*	
Abdominal wall/hernia surgery	508
Acute care/emergency general	440
Minimal invasive surgery	440
Colon and rectal surgery	412
Surgical oncology	251
Hepatopancreaticobiliary surgery	197
Trauma surgery	155
Breast surgery	149
Gastroenterology surgery	148
Endocrine surgery	131
Pediatric surgery	46
Vascular surgery	43
Other	77
Region, *n* (%)	
North America	564 (55.08%)
Europe	302 (29.49%)
Asia	116 (11.33%)
South America	21 (2.05%)
Africa	16 (1.56%)
Oceania	5 (0.49%)

We report the median with IQR in parentheses for continuous variables and count with the percent of the total in parentheses for categorical variables

^a^For academic role and specialty, we allowed participants to select multiple options. As a result, we are unable to calculate a true percentage. For any respondent who identified as a chair, vice chair, division chief, program director, assistant program director, or sustainability lead, we backfilled an identification of “Consultant/Attending” if not already selected

A slight majority of respondents (*n* = 518, 51%) practiced in academic hospitals, while fewer practiced in community or private teaching hospitals (*n* = 265, 26%) and community or private non-teaching hospitals (*n* = 121, 12%). Fewer still practiced in public access hospitals (*n* = 100, 10%), military hospitals (*n* = 15, 1.5%), or Veterans Affairs hospitals (*n* = 5, 0.5%). A large majority of respondents practiced in larger hospital settings, with 421 (41%) practicing in hospitals with 100 to 500 beds and 407 (40%) practicing in hospitals with greater than 500 beds.

We asked respondents to select the specialties in which they practiced, allowing for more than one selection if applicable. With this context, the most represented specialties were Abdominal Wall and Hernia Surgery (*n* = 508), Acute Care or Emergency General Surgery (*n* = 440), Bariatric or Minimally Invasive Surgery (*n* = 440), and Colon and Rectal Surgery (*n* = 412). We similarly allowed respondents to choose multiple roles within the hospital, if applicable. To facilitate our analysis, we assumed that respondents who chose senior roles such as “Chair” or “Program Director” were also attending surgeons and backfilled this option if not selected. In the updated dataset, most respondents were consultants or attending surgeons (*n* = 830), of which 159 were division chiefs, 149 were program or associate program directors, 142 were chairs or vice chairs, and 16 were sustainability leads. On the other hand, 167 respondents were trainees, of which 97 were residents or house staff, 68 were fellows, and two were medical students. Of note, four respondents listed both “resident” and “fellow” as roles. We reclassified these respondents as fellows alone to avoid double counting. Two respondents did not list a role, and 50 respondents listed a role of “Other.” Upon manual review of “Other” roles, most were attending surgeons, though some were research assistants, PhD students, or physicians’ assistants.

The median age of all respondents was 44 (IQR 37–54) while median years in practice was 10 (IQR 3–20).

### General attitudes toward sustainability

**Fig. 3:**
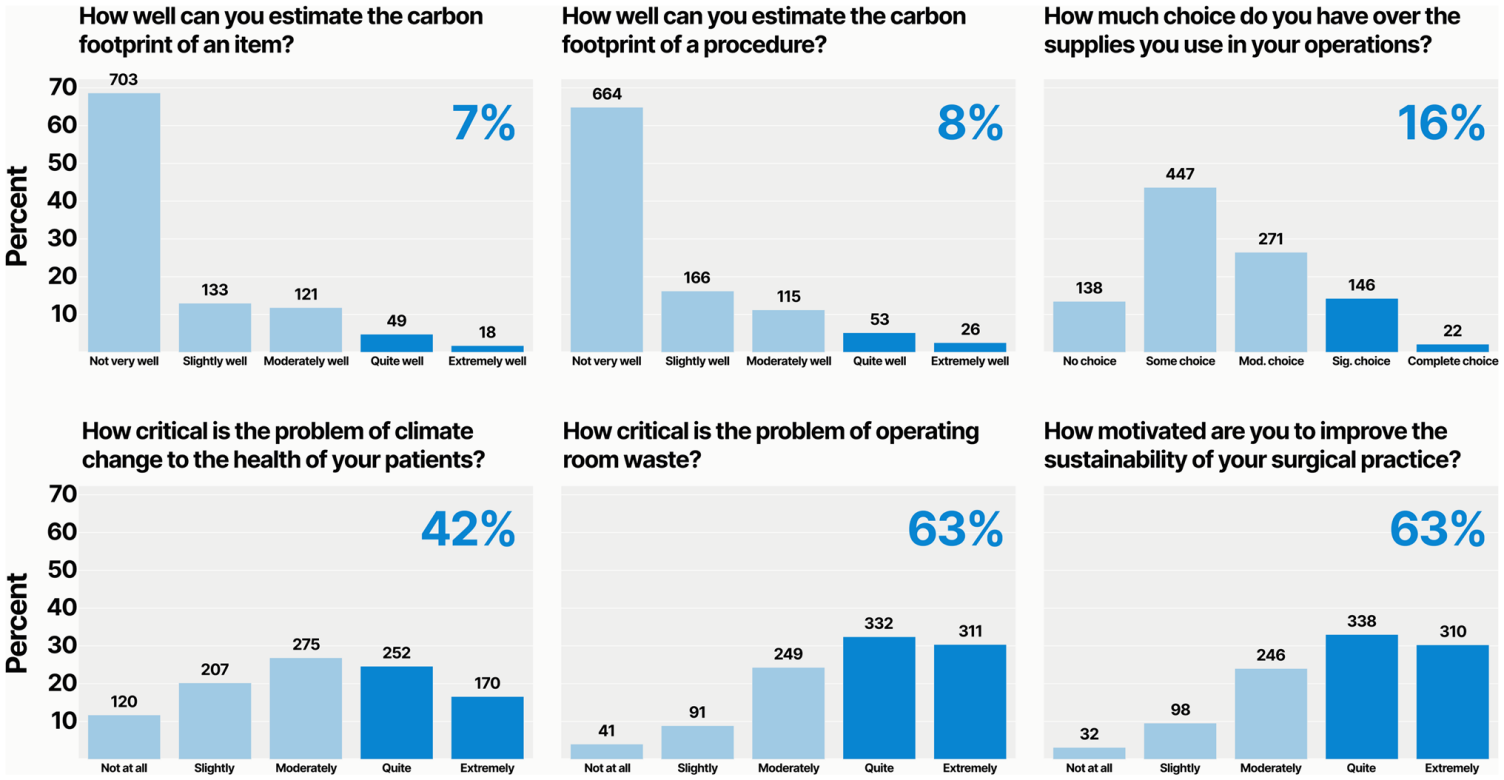
General attitudes and education levels about sustainability. The proportion of respondents who chose the top two Likert options is highlighted

Most respondents (*n* = 643, 63%) felt that operating room waste is a critical problem, and a similar number (*n* = 648, 63%) were motivated to improve the sustainability of their practice. On the other hand, fewer (*n* = 422, 41%) felt that climate change was a critical problem for the health of their patients. Of note, only 168 (16%) felt they had a choice in the supplies used during their operations (Fig. 3).

### Ability to estimate carbon footprint

When inquiring about respondents’ current knowledge about metrics used to measure sustainability, a small minority could estimate the carbon footprint of a surgical procedure (*n* = 79, 7.7%) or surgical supply (*n* = 67, 6.5%) (Fig. 3).

### Concerns about making sustainable changes

**Fig. 4:**
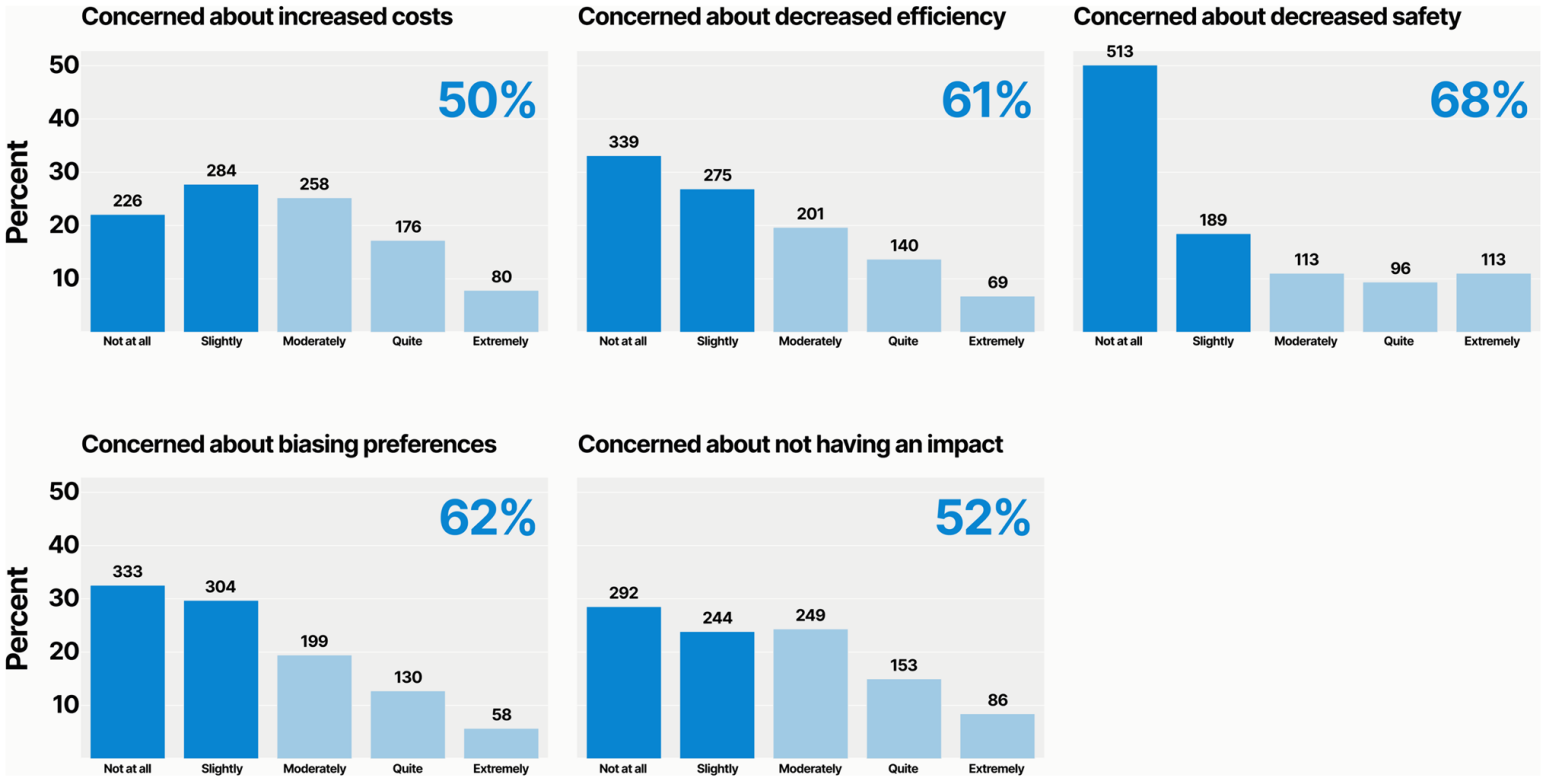
Concerns about the negative impacts of improving sustainability. The proportion of respondents who were “not at all” or “slightly” concerned is highlighted

We specifically asked respondents about their level of concern that improving operating room sustainability would increase costs, decrease efficiency, reduce safety, bias surgical preferences, or have no meaningful impact on climate change. We found that most respondents were “not at all” or “slightly” concerned about these consequences. Specifically, 510 (50%) had low levels of concern about increased costs, 620 (61%) about decreased efficiency, 700 (68%) about reduced safety, 630 (62%) about biased surgical preferences. When asking respondents whether sustainability efforts would be futile in impacting climate change, 520 (52%) expressed low levels of concern. Conversely, about half of respondents each harbor at least moderate concern that improving sustainability will increase costs or have no impact (Fig. 4).

### Willingness to change

**Fig. 5:**
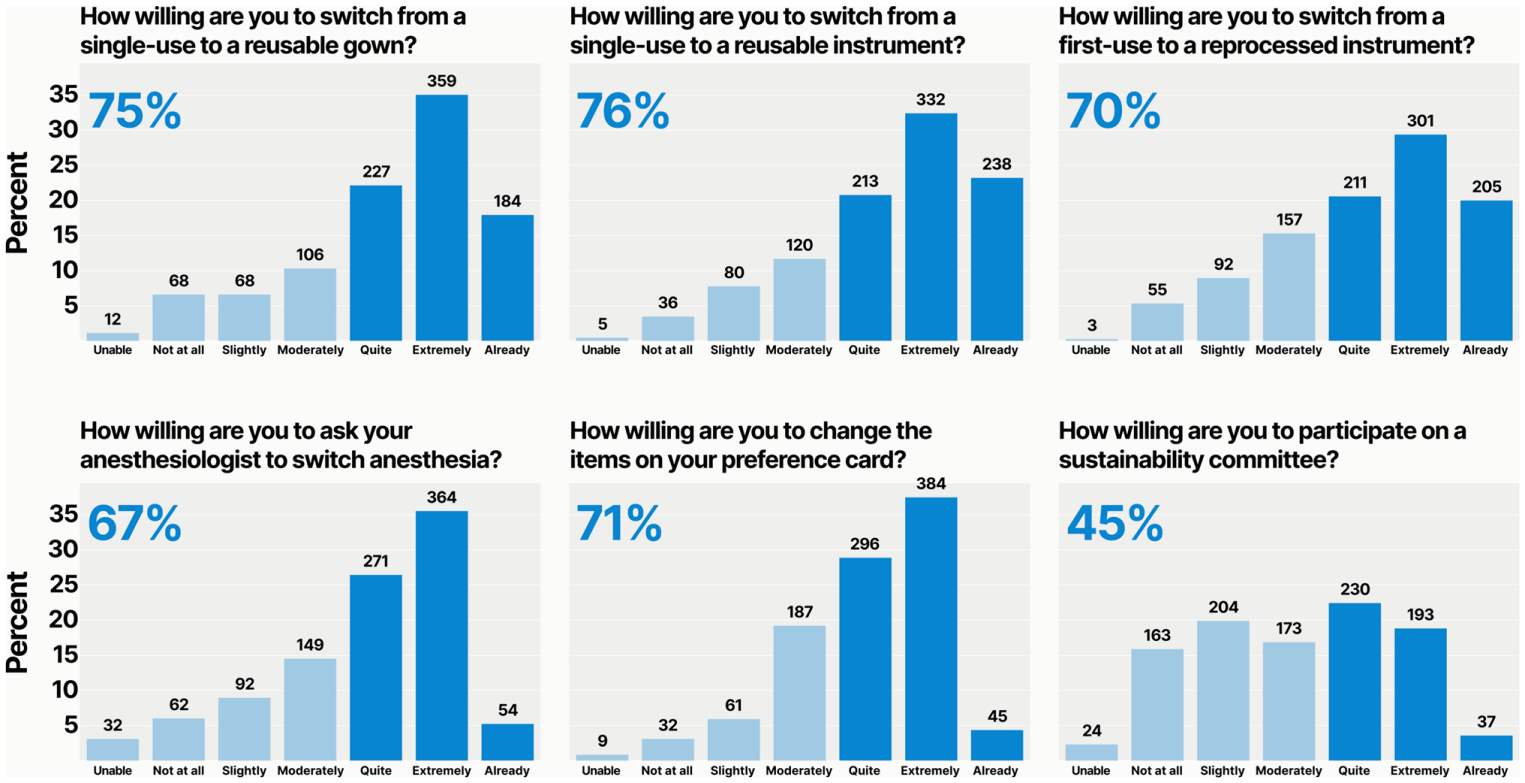
Willingness to make specific sustainable changes. The proportion of respondents who were “quite willing,” “extremely willing,” or “already doing” the proposed change is highlighted

Most surgeons expressed willingness to make each of the changes suggested, except giving up one hour per week to join a sustainability committee (*n* = 460, 45%). For example, 783 (77%) were willing to switch from a single-use instrument to a reusable instrument, 770 (75%) to switch from a single-use gown to a reusable gown, 725 (71%) to change the items on their preference cards to optimize carbon footprint, 717 (70%) to switch from a first-time use instrument to a reprocessed instrument, and 689 (67%) to ask the anesthesiologist to switch to a more sustainable anesthetic (Fig. 5).

### Preferred educational modalities

**Fig. 6:**
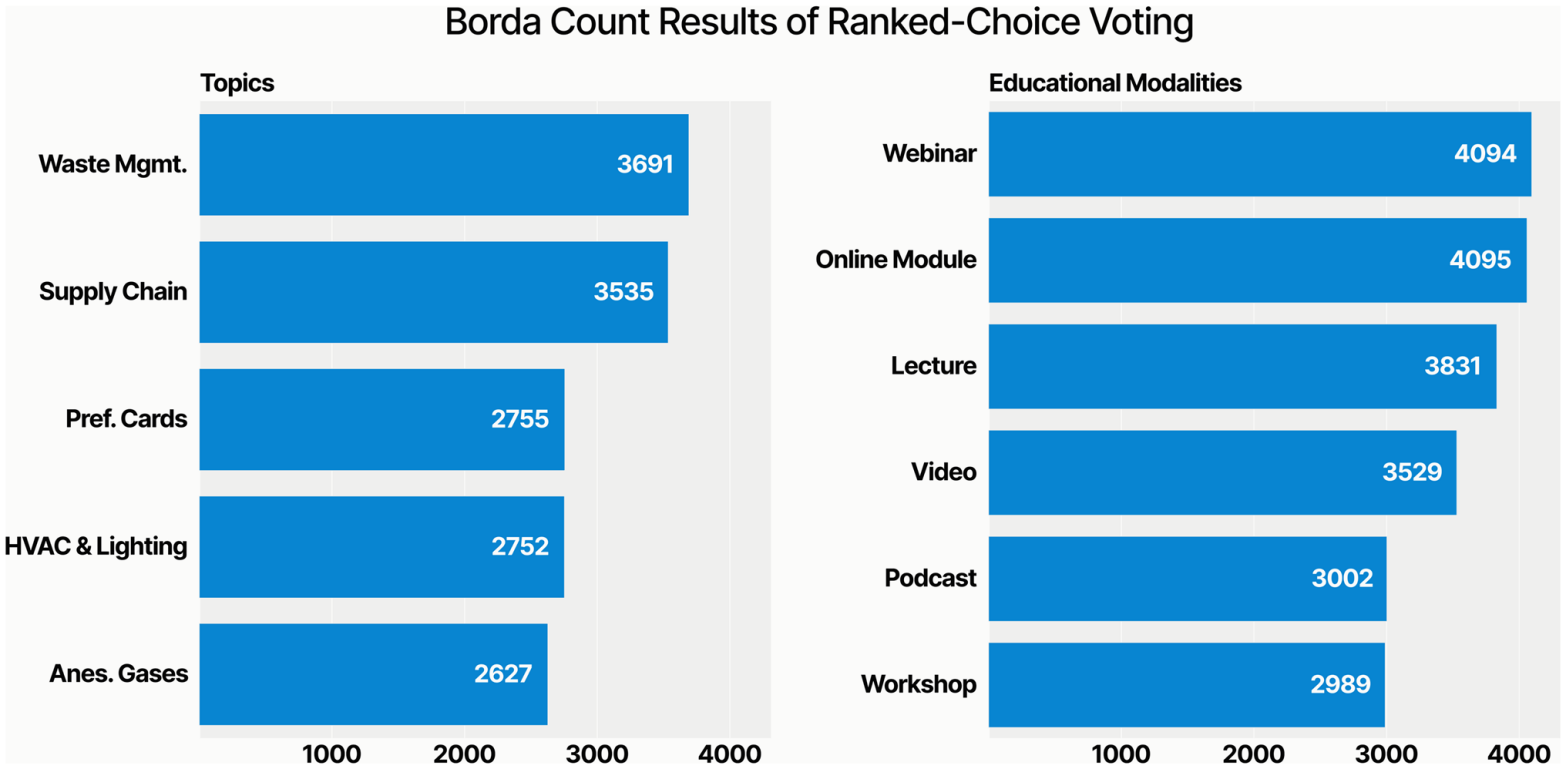
Ranked choice preferences of sustainability education topics and modalities. We used a Borda count methodology. For example, if there were five options, respondents received five points for their first choice, four points for their second choice, three points for their third choice, two points for their second choice, and one point for their fifth (last choice). We added point tallies for each option

Using Borda count voting, the most popular educational modality was an online webinar (4094 points), followed by an online module (4059 points), lecture (3851 points), educational video (3529 points), podcast (3002 points), and group workshop (2989 points). The most popular educational topic was waste management (3691 points), followed by supply chain (3535 points), preference card optimization (2755 points), heating, ventilation, and air conditioning (HVAC points) and lighting (2752 points), and anesthetic gasses (2627 points) (Fig. 6).

### Comparing results across demographic groups

Overall, North American respondents were less motivated to improve the sustainability of their practice, had less choice in selecting surgical supplies, were less able to estimate the carbon footprint of surgical procedures and supplies, and were less willing to join a sustainability committee than respondents from Europe or other regions. On the other hand, North American respondents were less concerned about the negative effects of improving sustainability than respondents from Europe or other regions. European respondents were most willing to adopt reusable or reprocessed instruments.

Respondents in leadership roles viewed the criticality of operating room waste to a lesser degree than their non leader counterparts. Respondents in leadership roles were also more likely to report being able to estimate the carbon footprint of surgical procedures and supplies and reported more choice in selecting surgical supplies. They were more concerned about the increased cost and decreased efficiency associated with sustainable changes and also more concerned that sustainable changes would have no impact. They were less willing to adopt reusable gowns.

Trainees were less likely to report being able to estimate the carbon footprint of surgical procedures and supplies as compared to non-trainees. They also reported less choice in selecting surgical supplies. They were more willing to adopt reusable instruments. Further data regarding the proportion of respondents expressing pro-sustainability views for each question, across different demographic groups, are provided in Supplementary Table 1.

### Cluster analysis

The K-means algorithm delineated three distinct clusters within our data. These clusters are visually represented in Supplementary Fig. 1, where they are plotted according to Principal Component 1 (PC1) and Principal Component 2 (PC2) (Supplementary Fig. 1). Though PCs do not independently have meaning, they help visualize the separation of respondents in our data. By measuring the relative contribution of each question to a PC, we can think of them as a weighted average variable representing multiple questions. In our case, PC1 is predominantly influenced by the general attitudes and willingness questions while PC2 is predominantly influenced by the concern questions.

Cluster 1 is characterized by the lowest levels of willingness, moderate concerns, and a less positive attitude in comparison to the other clusters. In contrast, Cluster 2 is distinguished by the highest levels of concern, greater willingness, and a more positive attitude. Cluster 3 stands out with the least concerns and the highest willingness (Supplementary Table 2). Comprehensive demographic details for each cluster are presented in Supplementary Table 3. Demographic differences between clusters can help inform future efforts by SAGES-EAES to provide tailored education and outreach related to sustainability.

## Discussion

In this survey, we demonstrated that a majority of surgeons view environmental sustainability in surgery as a critical problem and are motivated to improve it. On the other hand, less than half believe that climate change is a critical problem to the health of their patients and most are not able to estimate the carbon footprint of a surgical operation or supply. This highlights both a baseline interest in the topic of improving sustainability as well as the presence of a knowledge gap surrounding its specifics. Interestingly, surgeons are not overly concerned that improving sustainability will negatively impact cost, safety, efficiency, or their own autonomy. Finally, most are willing to adopt one or more well-established practices to improve sustainability.

Our study differs meaningfully from prior international studies that captured surgeons’ perspectives on sustainability. First, our study had more responses (*n* = 1024) than those conducted by the Association of Surgeons from Great Britain and Ireland (ASGBI) in 2020 (*n* = 130), the European Society of Coloproctology (ESCP) in 2022 (*n* = 370), the European Society of Gastrointestinal Endoscopy in 2023 (ESGE) (*n* = 407), and the World Society for Emergency Surgery (WSES) in 2023 (*n* = 450). Moreover, we surveyed surgeons from multiple specialties as opposed to the ESCP study and the WSES study, which surveyed Colorectal and Trauma surgeons, respectively. Though our respondents were predominantly from North America (55%), our survey featured substantial participation from Europe (29%), Asia (11%), and other areas. Thus, our study’s global representation contrasts with the more localized foci of the ASGBI study on the United Kingdom, the WSES study on Europe, and the ESCP and the ESGE studies on Europe and Asia [19, 22, 23].

While our results largely echo those from prior studies, there are notable differences in magnitude. On the one hand, our finding that six in ten respondents are motivated to improve the sustainability of their practice and between 45 and 76% are willing to make one of the specific changes proposed supports the above average agreement to the statement “Green management should be a priority in all medical specialties” in the WSES study (mean = 3.98 out of 5). On the other hand, this response rate is considerably less than the 82% and 97% ASGBI and ESCP respondents’ willingness to improve sustainability in surgical practice [21, 22]. Differences between our results and those from prior studies may relate to the specific ways our questions were worded, making direct comparison difficult. Moreover, the aforementioned differences in geographic and subspecialty sampling may account for some of these differences.

Regarding potential barriers, 32% of respondents in our study were concerned that improving sustainability would decrease safety. This is greater than the 23% of ASGBI study respondents who were concerned about the same. Moreover, 50% of our respondents were worried about increasing costs, greater than the 16% who felt cost was a barrier in the ESGE study [23]. Both the ESGE and the ESCP studies referenced a lack of knowledge or understanding of what sustainability in practice entails, as a barrier, supported by our finding that most surgeons could not estimate the carbon footprint of a surgical process or supply [22, 23].

Willingness to adopt various sustainable changes was evident in our survey. A majority were positively inclined to incorporate all of the proposed changes with the exception of joining a sustainability committee (45%). Our proportion of respondents who would be quite or extremely willing to switch to reusable instruments (72%) is lower than the proportion of surgeons who exhibited high willingness to adopt reusable laparoscopic ports (92%) in the ASGBI study [21].

Our study has notable limitations. First, our data are mostly representative of North America and Europe. Both regions include countries with fewer resource constraints. As such, attitudes toward sustainability among surgeons in these countries may meaningfully differ from surgeons for whom becoming sustainable is an urgent imperative, not just theoretically beneficial. Additionally, although wide distribution of the survey achieved a high number of responses, it precluded our ability to calculate a response rate which would have informed our assessment over selection bias. It is reasonable to imagine that survey non-respondents are less likely to significantly engage in sustainability initiatives. Important limitations inherent to all sustainability survey studies include the possibility of the Hawthorne effect or social desirability bias, as sustainability is typically considered a positive trait, and respondents may have been biased to indicate more pro-sustainability views than they actually believe.

Ultimately, it is important to interpret our results in the context of where we, as surgeons, are in the movement toward sustainable surgery. The IDEAL framework has been previously described to characterize the evolution of surgical innovations from conception to practice, with early ideation driven by innovators, exploration of the innovation driven by pioneers, refinement of the innovation driven by early adopters, and finally, eventual incorporation into established practice [27]. By our estimation, the movement to improve sustainability has been thus far led by innovators and pioneers. By measuring surgeon attitudes toward sustainability, we can better determine how best to cultivate early adopters and teach them how to implement sustainable changes.

As surgeons are at different stages in their acceptance of sustainability today, they may require tailored education and outreach. To that end, clustering analysis allows us to understand unique phenotypes among our respondents and can inform the design of future interventions. For example, our clustering analysis revealed one group of respondents who are especially willing to make sustainable changes and have low concern about negative consequences and another group of respondents who are less willing to make sustainable changes and have a moderate level of concern. A third group of respondents were willing to make changes but had concerns about the negative impact of those changes. Future surveys and focus groups may help determine how to best design educational approaches for each cluster. For example, surgeons generally unfavorable to SSP could be educated on the specific contributions of surgical practices on climate change. Surgeons with concerns about the risks of using reprocessed instruments, could be provided evidence in support of their safety and efficacy. Surgeons eager to improve the sustainability of their practice could then be provided with specific toolkits on implementing changes and perhaps a roadmap on how to organize and implement sustainable action on a wide scale within their institution. Future surveys will help us monitor surgeons’ attitudes toward sustainability and level of involvement in sustainable practices over time.

Sustainability is a global problem that will require local solutions. While national and international bodies can and should set ambitious sustainability targets, individual institutions will need to creatively determine how to achieve those targets within their own constraints. Moreover, institutions will have to do so while respecting the attitudes and preferences of their constituent surgeons. Understanding surgeons’ attitudes, knowledge gaps and willingness to adopt sustainable measures was an essential first step to identify obstacles and opportunities for broader action. As a next step, surgical societies need to develop educational programs and practical guidance on how to implement incremental changes aimed to reduce the environmental impact of surgical practice. To that end, SAGES and EAES will be building on this survey and the results of a systematic review of current sustainability metrics and studies within gastrointestinal surgery to craft a position paper with a call to action. Finally, by partnering with other stakeholders, surgical societies can become engaged in broader advocacy efforts. For example, SAGES recently became a signatory of the Department of Health and Human Services Climate pledge. Together, this study and others like it can lay a foundation for increased surgeon engagement in improving the sustainability of surgical practice.

## Supplementary Information

Below is the link to the electronic supplementary material.

Supplementary file1 (TIFF 7157 KB) Principal Component Analysis (PCA) Scatter Plot of K-Means Cluster Distribution. This scatter plot visualizes the segmentation of the dataset into three distinct clusters as identified by K-means clustering algorithm, post-PCA reduction. Each point represents an observation projected onto the first two principal components (PCA1 and PCA2), which explain the majority of the variance. The separation among clusters indicates differing group characteristics within the multidimensional data, simplified here for a two-dimensional representation.

Supplementary file2 (DOCX 26 KB)

Supplementary file3 (DOCX 17 KB)

Supplementary file4 (DOCX 18 KB)
