# Supplementary figures and images for: Perspectives on sustainability among surgeons: findings from the SAGES-EAES sustainability in surgical practice task force survey
Source: Surg Endosc. 2024 Aug 19;38(10):5803–14. doi: 10.1007/s00464-024-11137-7 (PMC11458713; doi:10.1007/s00464-024-11137-7)

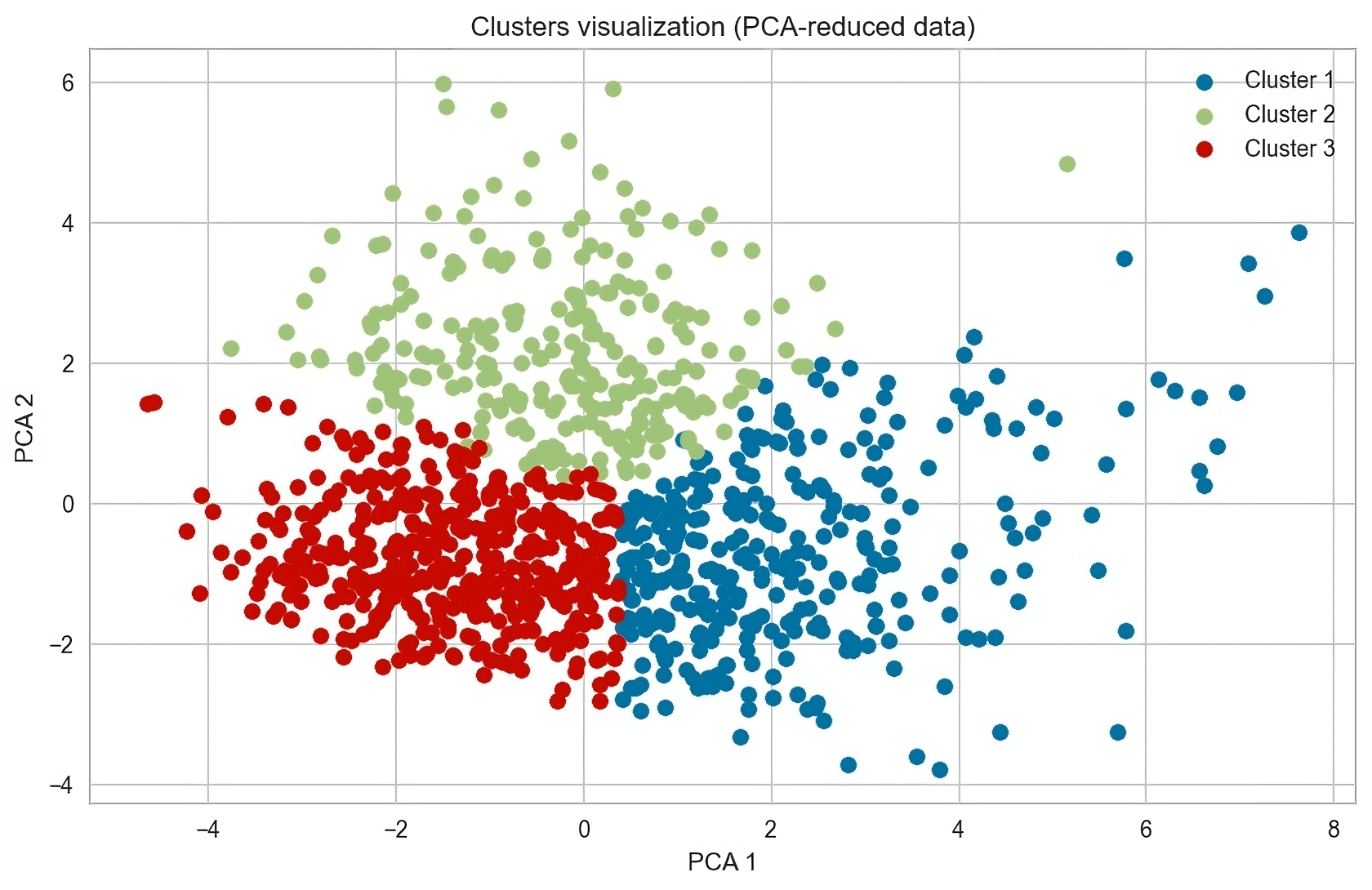

Supplement: Supplementary file 1 — Supplementary file1 (TIFF 7157 KB) Principal Component Analysis (PCA) Scatter Plot of K-Means Cluster Distribution. This scatter plot visualizes the segmentation of the dataset into three distinct clusters as identified by K-means clustering algorithm, post-PCA reduction. Each point represents an observation projected onto the first two principal components (PCA1 and PCA2), which explain the majority of the variance. The separation among clusters indicates differing group characteristics within the multidimensional data, simplified here for a two-dimensional representation. [file 464_2024_11137_MOESM1_ESM.tiff]
