# Supplementary material for: Perspectives on sustainability among surgeons: findings from the SAGES-EAES sustainability in surgical practice task force survey
Source: Surg Endosc. 2024 Aug 19;38(10):5803–14. doi: 10.1007/s00464-024-11137-7 (PMC11458713; doi:10.1007/s00464-024-11137-7)
Supplement: Supplementary file 2 — Supplementary file2 (DOCX 26 KB) [file 464_2024_11137_MOESM2_ESM.docx]

**Supplementary Table 1: Sustainability Views Among Respondent Demographic Groups**

|  | **Overall** | **Europe** | **North America** | **Other** | **P-Value** | **Non-leadership** | **Leadership** | **P-Value** | **Non-trainee** | **Trainee** | **P-Value** |
| --- | --- | --- | --- | --- | --- | --- | --- | --- | --- | --- | --- |
| Category (Question) | N = 1024 | N = 302 | N = 564 | N = 158 |  | N = 622 | N = 402 |  | N = 859 | N = 165 |  |
| Concern (increase costs) | 510 (49.8) | **138 (45.7)** | **322 (57.1)** | **50 (31.6)** | **<0.001** | **336 (54.0)** | **174 (43.3)** | **0.001** | 420 (48.9) | 90 (54.5) | 0.213 |
| Concern (decrease efficiency) | 614 (60.0) | **172 (57.0)** | **368 (65.2)** | **74 (46.8)** | **<0.001** | **391 (62.9)** | **223 (55.5)** | **0.022** | 507 (59.0) | 107 (64.8) | 0.189 |
| Concern (reduce safety) | 702 (68.6) | **190 (62.9)** | **432 (76.6)** | **80 (50.6)** | **<0.000** | 430 (69.1) | 272 (67.7) | 0.67 | 590 (68.7) | 112 (67.9) | 0.91 |
| Concern (bias preferences) | 637 (62.2) | **180 (59.6)** | **384 (68.1)** | **73 (46.2)** | **<0.001** | 395 (63.5) | 242 (60.2) | 0.318 | 525 (61.1) | 112 (67.9) | 0.12 |
| Concern (no impact) | 536 (52.3) | 147 (48.7) | 312 (55.3) | 77 (48.7) | 0.108 | **343 (55.1)** | **193 (48.0)** | **0.03** | 441 (51.3) | 95 (57.6) | 0.166 |
| Willingness (reusable gown) | 770 (75.2) | 228 (75.5) | 434 (77.0) | 108 (68.4) | 0.086 | **486 (78.1)** | **284 (70.6)** | **0.008** | 642 (74.7) | 128 (77.6) | 0.5 |
| Willingness (reusable instrument) | 783 (76.5) | **250 (82.8)** | **420 (74.5)** | **113 (71.5)** | **0.006** | 480 (77.2) | 303 (75.4) | 0.557 | **644 (75.0)** | **139 (84.2)** | **0.013** |
| Willingness (reprocessed instrument) | 717 (70.0) | **233 (77.2)** | **371 (65.8)** | **113 (71.5)** | **0.002** | 437 (70.3) | 280 (69.7) | 0.891 | 596 (69.4) | 121 (73.3) | 0.357 |
| Willingness (switch anesthesia) | 689 (67.3) | 214 (70.9) | 367 (65.1) | 108 (68.4) | 0.213 | 416 (66.9) | 273 (67.9) | 0.784 | 581 (67.6) | 108 (65.5) | 0.648 |
| Willingness (optimize PrefCard) | 725 (70.8) | 217 (71.9) | 403 (71.5) | 105 (66.5) | 0.423 | 440 (70.7) | 285 (70.9) | 0.99 | 608 (70.8) | 117 (70.9) | 0.99 |
| Willingness (join sustainability cmte) | 460 (44.9) | **180 (59.6)** | **183 (32.4)** | **97 (61.4)** | **<0.001** | 283 (45.5) | 177 (44.0) | 0.691 | 376 (43.8) | 84 (50.9) | 0.109 |
| Attitude (how much choice) | 168 (16.4) | **51 (16.9)** | **73 (12.9)** | **44 (27.8)** | **<0.001** | **78 (12.5)** | **90 (22.4)** | **<0.001** | **153 (17.8)** | **15 (9.1)** | **0.008** |
| Attitude (climate change critical) | 422 (41.2) | 110 (36.4) | 245 (43.4) | 67 (42.4) | 0.128 | 253 (40.7) | 169 (42.0) | 0.713 | 344 (40.0) | 78 (47.3) | 0.101 |
| Attitude  (waste) | 643 (62.8) | 201 (66.6) | 344 (61.0) | 98 (62.0) | 0.265 | **412 (66.2)** | **231 (57.5)** | **0.006** | 529 (61.6) | 114 (69.1) | 0.082 |
| Attitude (motivation) | 648 (63.3) | **232 (76.8)** | **308 (54.6)** | **108 (68.4)** | **<0.001** | 402 (64.6) | 246 (61.2) | 0.295 | 533 (62.0) | 115 (69.7) | 0.075 |
| Knowledge (estimate items) | 67 (6.5) | **24 (7.9)** | **19 (3.4)** | **24 (15.2)** | **<0.001** | **31 (5.0)** | **36 (9.0)** | **0.017** | **66 (7.7)** | **1 (0.6)** | **0.001** |
| Knowledge (estimate procedures) | 79 (7.7) | **27 (8.9)** | **22 (3.9)** | **30 (19.0)** | **<0.001** | **33 (5.3)** | **46 (11.4)** | **0.001** | **74 (8.6)** | **5 (3.0)** | **0.021** |

**Supplementary Table 1.** Proportion of respondents with pro-sustainability views across various demographic groups
