# Supplementary material for: Perspectives on sustainability among surgeons: findings from the SAGES-EAES sustainability in surgical practice task force survey
Source: Surg Endosc. 2024 Aug 19;38(10):5803–14. doi: 10.1007/s00464-024-11137-7 (PMC11458713; doi:10.1007/s00464-024-11137-7)
Supplement: Supplementary file 3 — Supplementary file3 (DOCX 17 KB) [file 464_2024_11137_MOESM3_ESM.docx]

**Supplementary Table 2: Cluster Analysis of Survey Respondents**

|  | Total | Cluster 1, | Cluster 2, | Cluster 3, | P-Value |
| --- | --- | --- | --- | --- | --- |
| Category (Question) | N = 1024 | N =321 | N =258 | N =445 |  |
| Concern  (increase costs) | 2.6 | 2.6 | 3.5 | 2 | <0.001 |
| Concern  (decrease efficiency) | 2.3 | 2.6 | 3.4 | 1.6 | <0.001 |
| Concern  (reduce safety) | 2.1 | 2.2 | 3.4 | 1.3 | <0.001 |
| Concern  (bias preferences) | 2.3 | 2.4 | 3.3 | 1.6 | <0.001 |
| Concern  (no impact) | 2.5 | 2.6 | 3.3 | 2 | <0.001 |
| Willingness  (reusable gown) | 4.2 | 3.3 | 4.5 | 4.7 | <0.001 |
| Willingness  (reusable instrument) | 4.4 | 3.5 | 4.8 | 4.8 | <0.001 |
| Willingness  (reprocessed instrument) | 4.2 | 3.2 | 4.6 | 4.6 | <0.001 |
| Willingness  (switch anesthesia) | 3.8 | 2.7 | 4.2 | 4.4 | <0.001 |
| Willingness  (optimize PrefCard) | 4 | 3 | 4.4 | 4.6 | <0.001 |
| Willingness  (join sustainability cmte) | 3.1 | 1.9 | 3.9 | 3.5 | <0.001 |
| Attitude  (how much choice) | 2.5 | 2.3 | 2.9 | 2.4 | <0.001 |
| Attitude  (climate change critical) | 3.1 | 2.2 | 3.4 | 3.6 | <0.001 |
| Attitude  (waste) | 3.8 | 2.9 | 4 | 4.2 | <0.001 |
| Attitude  (motivation) | 3.8 | 2.8 | 4.1 | 4.3 | <0.001 |
| Knowledge  (estimate items) | 1.6 | 1.4 | 2.2 | 1.4 | <0.001 |
| Knowledge  (estimate procedures) | 1.6 | 1.4 | 2.3 | 1.4 | <0.001 |

**Supplementary Table 2.** Comparative Analysis of Survey Question Responses Across Identified Clusters. Boxes represent mean scores for each question. Questions on attitudes and willingness were rated on a scale of 1 to 5, while questions on willingness were rated on a scale of 1 to 7. Darker shades represent higher mean responses.
