# Supplementary material for: Perspectives on sustainability among surgeons: findings from the SAGES-EAES sustainability in surgical practice task force survey
Source: Surg Endosc. 2024 Aug 19;38(10):5803–14. doi: 10.1007/s00464-024-11137-7 (PMC11458713; doi:10.1007/s00464-024-11137-7)
Supplement: Supplementary file 4 — Supplementary file4 (DOCX 18 KB) [file 464_2024_11137_MOESM4_ESM.docx]

**Supplementary Table 3: Demographic Differences Between Clusters**

|  |  | Overall  N = 1024 | Cluster 1  N = 321 | Cluster 2  N = 258 | Cluster 3  N = 445 | P-value |
| --- | --- | --- | --- | --- | --- | --- |
| Age, mean (SD) | | 45.8 (11.4) | 46.2 (11.9) | 46.6 (11.0) | 45.0 (11.2) | 0.153 |
| Years in practice, mean (SD) | | 12.7 (10.8) | 13.6 (11.2) | 13.8 (10.7) | 11.5 (10.6) | 0.005 |
| Region, n (%) | Africa | 16 (1.6) | 3 (0.9) | 6 (2.3) | 7 (1.6) | <0.001 |
|  | Asia | 116 (11.3) | 41 (12.8) | 56 (21.7) | 19 (4.3) |  |
|  | Europe | 302 (29.5) | 68 (21.2) | 105 (40.7) | 129 (29.0) |  |
|  | North America | 564 (55.1) | 205 (63.9) | 79 (30.6) | 280 (62.9) |  |
|  | Oceania | 5 (0.5) | 1 (0.3) | 3 (1.2) | 1 (0.2) |  |
|  | South America | 21 (2.1) | 3 (0.9) | 9 (3.5) | 9 (2.0) |  |
| Practice setting, n (%) | Academic Hospital | 518 (50.6) | 149 (46.4) | 122 (47.3) | 247 (55.5) | 0.016 |
|  | Community or Private Non-Teaching Hospital | 121 (11.8) | 51 (15.9) | 23 (8.9) | 47 (10.6) |  |
|  | Community or Private Teaching Hospital | 265 (25.9) | 89 (27.7) | 76 (29.5) | 100 (22.5) |  |
|  | Military Hospital | 15 (1.5) | 5 (1.6) | 3 (1.2) | 7 (1.6) |  |
|  | Public Access Hospital | 100 (9.8) | 26 (8.1) | 34 (13.2) | 40 (9.0) |  |
|  | Veterans Affairs Hospital | 5 (0.5) | 1 (0.3) |  | 4 (0.9) |  |
| Practice size, n (%) | 100 and 500 beds | 421 (41.1) | 138 (43.0) | 100 (38.8) | 183 (41.1) | 0.001 |
|  | Between 25 and 50 beds | 151 (14.7) | 51 (15.9) | 55 (21.3) | 45 (10.1) |  |
|  | Fewer than 25 beds | 45 (4.4) | 16 (5.0) | 14 (5.4) | 15 (3.4) |  |
|  | Greater than 500 beds | 407 (39.7) | 116 (36.1) | 89 (34.5) | 202 (45.4) |  |
| Trainee status, n (%) | Trainee | 165 (16.1) | 47 (14.6) | 38 (14.7) | 80 (18.0) | 0.363 |
|  | Non-trainee | 859 (83.9) | 274 (85.4) | 220 (85.3) | 365 (82.0) |  |
| Leadership status, n (%) | Leadership | 402 (39.3) | 129 (40.2) | 118 (45.7) | 155 (34.8) | 0.016 |
|  | Non-leadership | 622 (60.7) | 192 (59.8) | 140 (54.3) | 290 (65.2) |  |

**Supplementary Table 3.** Comparative Overview of Demographic Characteristics Across Identified Clusters
